# Current Evidences and Future Perspectives for AMPK in the Regulation of Milk Production and Mammary Gland Biology

**DOI:** 10.3389/fcell.2020.00530

**Published:** 2020-06-26

**Authors:** Zhihui Wu, Min Tian, Jinghui Heng, Jiaming Chen, Fang Chen, Wutai Guan, Shihai Zhang

**Affiliations:** ^1^Guangdong Provincial Key Laboratory of Animal Nutrition Control, College of Animal Science, South China Agricultural University, Guangzhou, China; ^2^College of Animal Science and National Engineering Research Center for Breeding Swine Industry, South China Agricultural University, Guangzhou, China; ^3^Guangdong Laboratory for Lingnan Modern Agriculture, South China Agricultural University, Guangzhou, China; ^4^Department of Molecular Biology, University of Texas Southwestern Medical Center, Dallas, TX, United States

**Keywords:** mammary epithelial cells, AMPK, milk fat, milk protein, lactose

## Abstract

Activated protein kinase (AMP)-activated protein kinase (AMPK) senses the cellular energy status and coordinates catabolic and anabolic processes. Extensive studies have proposed that AMPK regulates energy homeostasis, cell growth, autophagy, mitochondrial biology and inflammation. The biological functions of AMPK vary in different tissues or organs. As a unique organ that produces milk, the mammary gland has recently attracted substantial research attention. This review discusses how AMPK in the mammary gland is activated by energy deprivation and heat stress via the activation of canonical and non-canonical pathways. In addition, the important downstream targets of AMPK and their functions in the mammary gland, especially during milk synthesis, are summarized in the review.

## Introduction

Energy homeostasis plays a critical role in maintaining the survival and function of cells. In 1967, Atkinson proposed using the adenylate system [ATP (adenosine 5′ triphosphate) + ADP (adenosine diphosphate) + AMP (adenosine monophosphate)] to estimate cellular energy levels (Atkinson and Fall, 1967; Atkinson and Walton, 1967). ATP acts as a high-energy bond donor, and ATP can be broken down into ADP and AMP, which is accompanied by the release of energy. Increased levels of cellular ADP and/or AMP are indicators of decreased energy status. AMP-activated protein kinase (AMPK) is the dominant energy sensor in eukaryotic cells (Hardie, 2007). As a master regulator of metabolism, AMPK is proposed to regulate energy homeostasis (Hardie et al., 2012), cell growth (Mihaylova and Shaw, 2011), autophagy (Mihaylova and Shaw, 2011), and mitochondrial biology (Herzig and Shaw, 2018). Recent advances in research have proposed that the biological effects of AMPK can be tissue- and/or cell type-specific. Hypothalamic AMPK modulates the energy balance of the whole body via the regulation of food intake (López et al., 2016). In T cells, AMPK regulates cell migration and determines CD8+ T cell fate (differentiation into effector or memory CD8+ T cells) (Finlay and Cantrell, 2011). However, the functions of AMPK in the mammary gland are still unclear.

The mammary gland is a unique organ for the secretion of milk. The mammary gland contains not only the epithelial cells but also the stromal compartment (also known as connective tissue or fat pad) (Hennighausen and Robinson, 2005). Mammary epithelium includes luminal and secretory cells that are involved in milk production during lactation period. The primary components of the mammary stroma are adipocytes, but it also contains fibroblasts, blood vessels and neurons. Milk directly regulates the early growth and health of neonates. The main components of milk are carbohydrates, lipids, and proteins with small proportions of minerals and vitamins. Colostrum is the milk secreted shortly after fallowing and consists a higher level of immunoglobulins (IgG, IgM, and IgA). These immunoglobulins are involved in the establishment of passive immunity for neonates. The energy requirements and mobilization in the mammary gland are high and very complicated. During lactation, energy is not only consumed by the mammary gland to maintain its basic metabolism but also largely used for milk production (Sampson and Jansen, 1984). In this review, we summarized recent advances in the understanding of the mechanism by which AMPK regulates milk production and mammary gland biology. Understanding the functions of AMPK and its downstream targets in the mammary gland will contribute to the development of nutritional or drug-related strategies for improving mammary gland health and promoting milk production.

## AMP-Activated Protein Kinase

AMP-activated protein kinase exists as a heterotrimer, which consists of one catalytic subunit (α) and two regulatory subunits (β and γ) (Xiao et al., 2011). There are multiple isoforms of these AMPK subunits, including α1, α2, β1, β2, γ1, γ2, and γ3 (Hardie et al., 2006). AMPKα1 is dominantly expressed in the mammary gland (Quentin et al., 2011), while AMPK α2 and γ3 are undetectable (McFadden and Corl, 2009). This evidence indicates that the expression of AMPK subunits could be tissue dependent.

The canonical mechanism by which AMPK is activated (phosphorylation at Thr172) relies on increased levels of ADP, AMP or Ca^2+^. LKB1 (liver kinase B1) and CaMKKβ (Ca^2+/^calmodulin-activated protein kinase kinase-β) are the primary upstream kinases of AMPK (Hawley et al., 2003, 2005). During conditions of energy deprivation, ADP can directly bind to the γ regulatory subunit of AMPK (Hardie et al., 2012). In even more severe situations, ADP is converted to AMP, which leads to a conformational change in AMPK and increases its activity by approximately 10-fold (Hardie et al., 2012). This conformational change also protects AMPK from being dephosphorylated and ensures its activation. In addition, increased levels of Ca^2+^ can directly regulate AMPK activity through CaMKKβ and in manner that is independent of the level of ADP or AMP (Hawley et al., 2005). In the human mammary gland, activated LKB1 and CaMKKβ are also reported to increase the activity of AMPK (Høyer-Hansen et al., 2007; Chung et al., 2017).

In addition to ADP, AMP and Ca^2+^, AMPK can also be activated by non-canonical signals, such as low oxygen (Marsin et al., 2000), reactive oxygen species (ROS) (Alexander et al., 2010; Ditch and Paull, 2012) and DNA damage (Ji et al., 2010; Sanli et al., 2010). When oxygen is limited, nutrients are not completely oxidized, which limits the production of ATP (Depré et al., 1998). ROS are proposed to regulate AMPK through two different pathways: (1) oxidation of the two conserved Cys residues in the AMPK α subunit (Alexander et al., 2010) and (2) activation of ataxia telangiectasia mutated (ATM), which is a phosphoinositide 3 kinase-like kinase (PIKK) that triggers the activation of AMPK (Ditch and Paull, 2012). DNA damage-induced activation of AMPK is also ATM-dependent (Storozhuk et al., 2013; Tripathi et al., 2013).

## Activation of AMPK in the Mammary Gland

**FIGURE 1:**
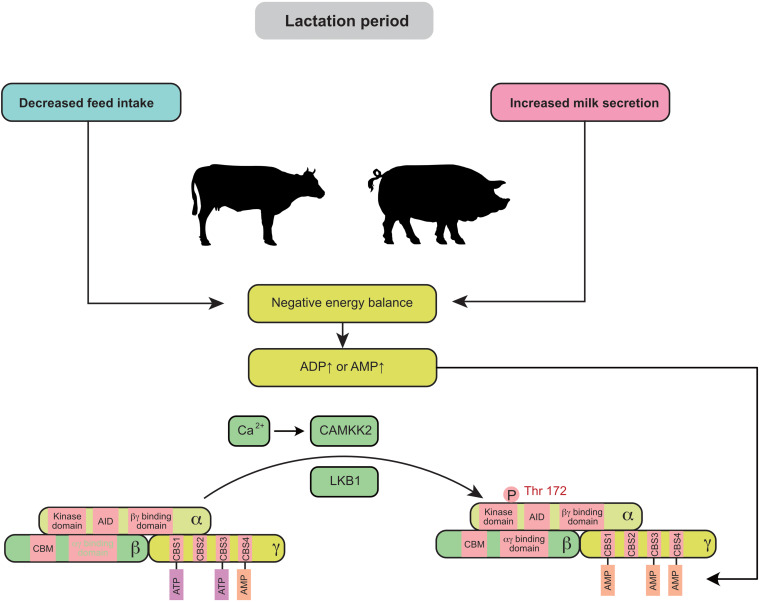
Negative energy balance induces the activation of mammary AMPK through the canonical pathway: during lactation, decreased maternal feed intake usually fails to meet the requirement for milk secretion and leads to a negative energy balance. Elevated ADP or AMP in the mammary gland is associated with a negative energy balance and promotes AMPK activity. AID, auto-inhibitory domain; CAMKK2, calmodulin dependent protein kinase kinase 2; CBM, carbohydrate-binding module; CBS, cystathionine-beta-synthase; LKB1, liver kinase B1.

Negative energy balance (NEB) is widely observed in swine and cow production, especially during lactation. Elevated ADP or AMP is often associated with a NEB (Figure 1). The feed intake of primiparous sows usually fails to meet the nutritional requirements for fetal growth and milk production, leading to the mobilization of protein and fat reserves in the body. Similarly, the peak lactation period of cows generally occurs 4–6 weeks after delivery, but the dry matter intake (DMI) does not reach its maximum until 8–10 weeks after delivery (Bertics et al., 1992). Thus, during this period, the energy intake in dairy cows is not able to meet the demand for milk production (especially in high-yielding cows). AMPK signaling in the mammary gland is highly active during this period, which results in a 15–20% decrease in milk production in sows and cows (Eastham et al., 1988; Wang et al., 2018). *In vitro* experiments also demonstrated that AMPK is significantly activated in mammary epithelial cells during energy deprivation (cells cultured without glucose or amino acids) (Zhang M. et al., 2018).

**FIGURE 2:**
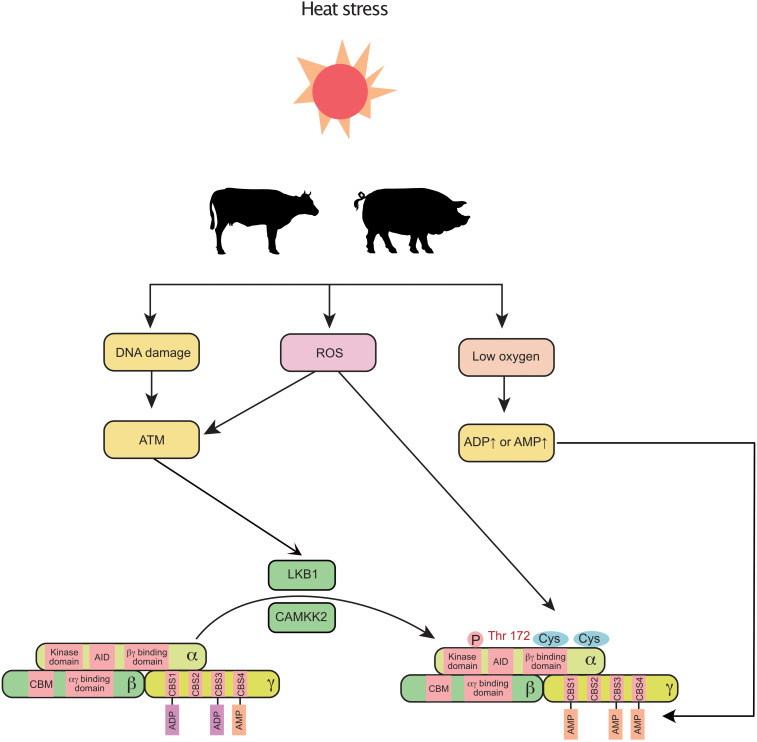
Heat stress induces the activation of mammary AMPK through canonical and non-canonical pathways: heat stress increases ROS, decreases blood oxygen, and alters DNA integrity, which further activates AMPK (non-canonical pathway). Additionally, the decreased feed intake (increased ADP and AMP) caused by heat stress also activates AMPK. ROS, reactive oxygen species; AID, auto-inhibitory domain; ATM, ataxia telangiectasia-mutated gene; CAMKK2, calmodulin dependent protein kinase kinase 2; CBM, carbohydrate-binding module; CBS, cystathionine-beta-synthase; LKB1, liver kinase B1.

In addition to NEB, heat stress is reported to negatively regulate mammary gland development and milk production in cows (Tao et al., 2011) and sows (Renaudeau and Noblet, 2001). Intriguingly, recent findings indicate that heat stress triggers the activation of AMPK in the mammary gland. In the murine mammary gland, the AMPK signaling pathway is significantly upregulated by heat stress (Han et al., 2019). In a transcriptomic study of the bovine mammary gland, AMPK signaling was the most highly activated pathway in response to heat stress (Gao et al., 2019). The non-canonical pathway could be a potential link between heat stress and AMPK (Figure 2). First, under heat stress, ROS are increased and accumulate in the bovine mammary gland (Li et al., 2019). Activation of AMPK decreases the production of ROS (El-Sisi et al., 2019) and enhances the antioxidant capacity (Guo et al., 2020) of the mammary gland. Second, oxygen uptake is significantly decreased in sows during heat stress (Black et al., 1993). Third, heat stress induces DNA damage in the mammary gland (Nair et al., 2010). In addition, heat stress also decreases feed intake in mammals, which indirectly triggers a decrease in energy intake and subsequently increases the levels of ADP and AMP. Therefore, heat stress can coordinately regulate AMPK through canonical and non-canonical pathways in the mammary gland.

## AMPK Regulates Milk Synthesis

### Milk Fat

**FIGURE 3:**
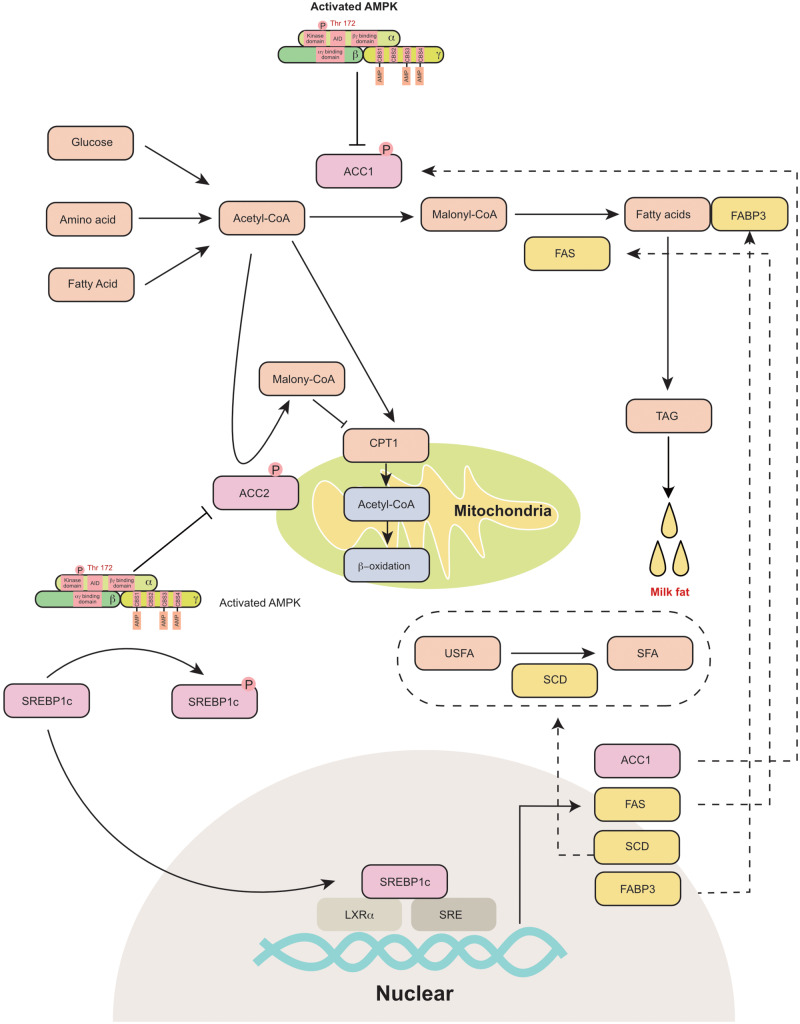
AMPK regulates mammary milk fat synthesis: AMPK phosphorylates and inactivates ACC1 and ACC2. ACC1 is a cytosolic protein that converts acetyl-CoA to malonyl-CoA during *de novo* fatty acid synthesis. ACC2 is associated with mitochondria and regulates mitochondrial fatty acid oxidation through the inhibition of CPT1 by malonyl-CoA. AMPK inhibits the transcriptional activity of SREBP-1c through the phosphorylation of SERBP1c at Ser372, which further decreases the expression of ACC1, FAS, SCD and FABP3, which participate in fatty acid synthesis. ACC1, acetyl-coA carboxylase 1; ACC2, acetyl-coA carboxylase 2; AID, auto-inhibitory domain; CBS, cystathionine-beta-synthase; CPT1, carnitine palmitoyl transferases 1; FABP3, fatty acid binding protein 3; FAS, fatty acid synthase; LXRα, liver X receptor α; SCD, stearoyl-CoA desaturase; SFA, saturated fatty acid; SRE, sterol-regulatory element; SREBP1c, sterol-regulatory element binding protein 1c; TAG, triacylglyceride; USFA, unsaturated fatty acid.

The process of milk fat synthesis in different species has been previously well summarized (Bionaz and Loor, 2008; Osorio et al., 2016; Zhang S. et al., 2018). Briefly, the process includes *de novo* fatty acid (FA) synthesis, FA uptake, FA activation, FA intracellular transport, FA elongation, FA desaturation, triacylglycerol (TAG) synthesis and lipid droplet formation. The FAs used for milk fat synthesis are either derived from blood circulation or are originally synthesized in the mammary gland. AMPK is a critical sensor that regulates fat metabolism in the mammary gland (Figure 3). It has been reported that AMPK activators 5-aminoimidazole-4-carboxamide 1-β-D-ribofuranoside (AICAR) and A-769662 (A76) are reported to inhibit fat synthesis in the bovine mammary gland (McFadden and Corl, 2009; Huang et al., 2020).

AMPK phosphorylates its downstream proteins at Ser/Thr residues (recognition motifs of AMPK: Φ(X,β)XXS/TXXXΦ) (Φ, hydrophobic; β, basic) (Dale et al., 1995). Acetyl-coA carboxylase (ACC) is a rate-limiting enzyme in fatty acid metabolism. It is one of the primary downstream pathways of AMPK. AICAR activates AMPK and inhibits ACC1 in goat mammary epithelial cells (Zhang et al., 2011). The coding and amino acid sequences of ACC were first identified in the murine mammary gland in 1988 (López-Casillas et al., 1988). Two isoforms of ACC have been identified in animals, namely, ACC1 (ACACA) and ACC2 (ACACB). ACC1 is a cytosolic protein that catalyzes the conversion of acetyl-CoA to malonyl-CoA during *de novo* FA synthesis (Abu-Elheiga et al., 2000). ACC2 is associated with the outer membrane of mitochondria. The malonyl-CoA produced by ACC2 regulates mitochondrial fatty acid oxidation through the inhibition of carnitine palmitoyltransferase 1 (CPT1) (Abu-Elheiga et al., 2000). ACC is highly expressed in the mammary gland during the lactation period (López-Casillas et al., 1991). The dominant isoform of ACC in the mammary gland varies among different animals. In the rat mammary gland, the main type of ACC is ACC2, while ACC1 is mainly expressed in white adipose tissue (López-Casillas et al., 1991; Ponce-Castañeda et al., 1991). However, both ACC1 and ACC2 are highly expressed in the murine mammary gland (Takebe et al., 2009). Similarly, ACC1 (Lv et al., 2015) and ACC2 (Palombo et al., 2018) are also detected in the porcine mammary gland and are upregulated during lactation. In dairy cows, ACC1 is considered a critical regulator of fatty acid synthesis in the mammary gland (Matsumoto et al., 2012; Tao et al., 2015). Short-term energy deprivation induces ACC phosphorylation and inhibits fatty acid synthesis in bovine mammary gland epithelial cells (McFadden and Corl, 2009). Thus, AMPK is proposed to control the switch between lipogenesis and lipid oxidation through the phosphorylation or dephosphorylation of ACCs.

The other critical downstream target of AMPK is sterol regulatory element-binding protein 1c (SREBP1c), which is a lipogenic transcription factor. Previously, AMPK activation (triggered by AICAR or A-769662) was reported to decrease the mRNA expression of lipogenic genes, including FAS and FABP3 in the bovine mammary gland (Zhang et al., 2011; Huang et al., 2020). AMPK inhibits the transcriptional activity of sterol regulatory element binding protein-1c (SREBP-1c) through the phosphorylation of SERBP1c at Ser372 (Li et al., 2011). SREBP1c was initially identified as a regulator of lipogenesis in the liver (Horton et al., 2002). Subsequently, SREBP1c has been proposed to regulate milk fat synthesis in cows (Li et al., 2014), goats (Xu et al., 2016b), and swine (Che et al., 2019). SREBP-1c binds to the promoter of lipogenic genes and increases their mRNA expression (Horton et al., 1998). The core promoter region of SREBP1c in the goat mammary gland is located from −395 to +1 bp upstream of the transcriptional start site and includes liver X receptor α (LXRα) binding elements and sterol regulatory elements (Xu et al., 2016a). In bovine mammary epithelial cells, the overexpression of SREBP1c significantly increases the expression of ACC1, FAS (fatty acid synthase), SCD (stearoyl-CoA desaturase) and FABP3 (fatty acid-binding protein) (Li et al., 2014). Consistently, the knockdown of SREBP1c decreases the expression of lipogenic genes (Li et al., 2014). In goat mammary epithelial cells, SREBP1c overexpression upregulates numerous genes that are responsible for *de novo* fatty acid synthesis and fatty acid transportation (Xu et al., 2016b). In the murine mammary gland, the knockdown of SREBP1c decreases the expression of FAS and SCD2 (Rudolph et al., 2010). Thus, AMPK can indirectly control transcription of lipogenic genes in the mammary gland through the phosphorylation of SREBP1c.

In addition to ACC and SREBP-1c, AMPK also regulates milk fat synthesis through adipose triglyceride lipase (ATGL) and TBC1 domain family member 1 (TBC1D1). ATGL mainly regulates the first step of the lipid lipolysis process. ATGL is a downstream target of peroxisome proliferator-activated receptor γ (PPARγ) (Shi et al., 2013) but not of SREBP1c (Xu et al., 2016b). Recently, AMPK was reported to activate ATGL through the phosphorylation of Ser406 (Ahmadian et al., 2011; Kim et al., 2016). In the goat mammary gland, ATGL is considered an important enzyme that regulates milk fat synthesis (59). In contrast to ATGL, TBC1D1 has been shown to regulate the translocation of glucose transporter 4 (GLUT4), which is an important insulin-sensitive transporter of glucose (Sakamoto and Holman, 2008). AMPK regulates the activity of TBC1D1 through the phosphorylation of Ser237 and Thr596 (Pehmøller et al., 2009). Interestingly, a whole genome resequencing study in cows showed that TBC1D1 expression is correlated with milk fat percentage (Jiang et al., 2016, 2019).

It is worth noting that some potential targets of AMPK might also regulate milk fat synthesis in the mammary gland. For example, AMPK increases the activity of Coactivator 1 alpha (PGC-1α) through the phosphorylation of Thr177 and Ser538 (Jäger et al., 2007). PGC-1α is a transcriptional coactivator of transcription factor PPARγ (Handschin and Spiegelman, 2006). In the liver, PGC-1α is proposed to regulate fatty acid β-oxidation through PPARγ (Inagaki et al., 2007). The other potential target protein is Liver X receptors (LXRs), which regulates the expression of SREBP1c and the process of lipogenesis. In the liver, AMPK is found to inhibit the ligand-induced LXR activity on Srebp-1c promoter (Yap et al., 2011). Intriguingly, recent studies reported that the activation of LXRs also increases the fatty acid synthesis in the mammary gland (McFadden and Corl, 2010; Oppi-Williams et al., 2013). To date, PGC-1α and LXR have been identified as downstream targets of AMPK in the liver. It would be interesting to know whether these mechanisms could be validated in the mammary gland as well.

### Milk Protein

Milk proteins, such as caseins and whey proteins, are important sources of nutrition for neonates. Colostrum contains immunoglobulins, such as IgG, IgM, and IgA, which are important to establish the early immunity of neonates. The amino acids used for milk protein synthesis are transported via a complicated transportation system (Zhang Y. et al., 2018). The process of milk protein synthesis requires a significant amount of energy (Moe, 1981). The master regulator of protein synthesis is mammalian target of rapamycin complex 1 (mTORC1). Dietary energy deprivation activates AMPK and inhibits mTORC1 in the rat mammary gland (Jiang et al., 2008). Similarly, in bovine mammary epithelial cells, energy deprivation increases the phosphorylation of AMPK by inhibiting the mTORC1 signaling pathway (Burgos et al., 2013). Glucose deprivation in bovine mammary epithelial cells leads to the downregulation of the mRNA expression of Casein Alpha S1 (CSN1S1), CSN2 and CSN3, which encode a_s1_-casein, β-casein and κ-casein, respectively (Zhang M. et al., 2018). However, glucose promotes αS1-casein and β-casein protein synthesis in the bovine mammary gland (Wang et al., 2019).

**FIGURE 4:**
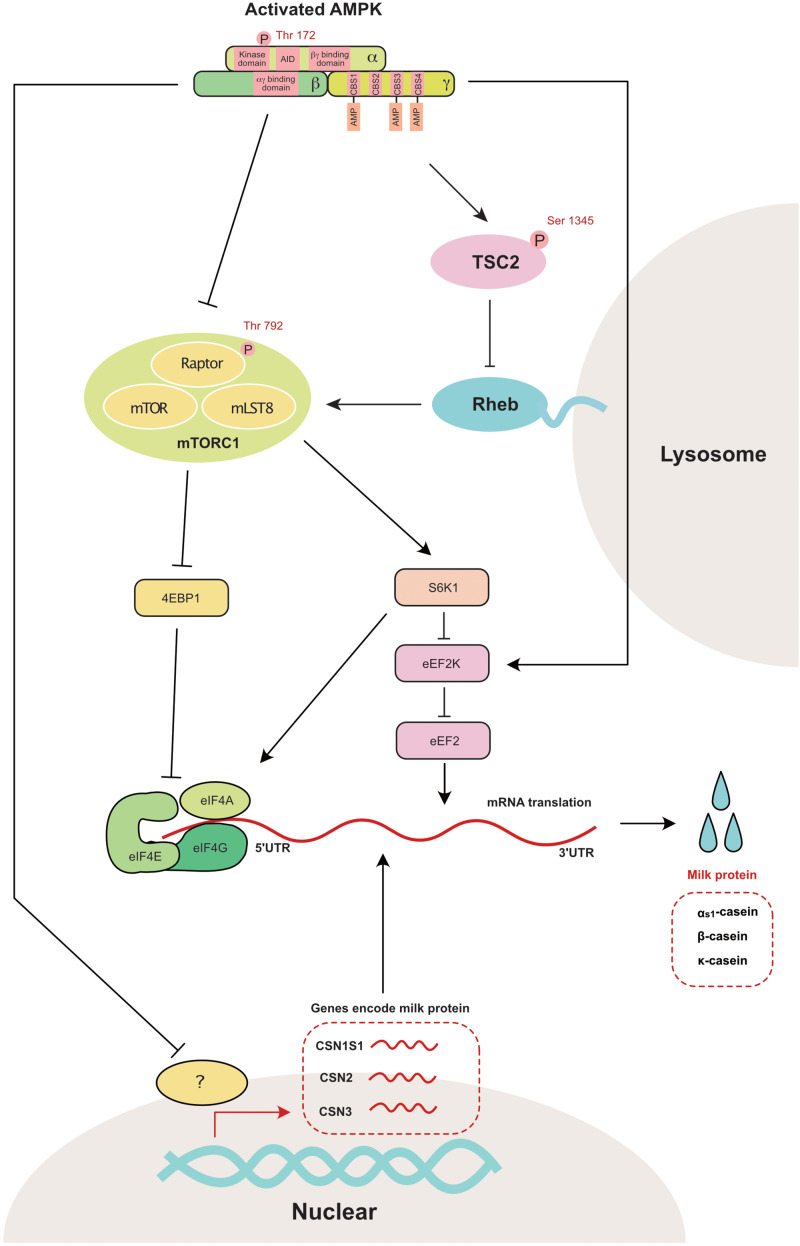
AMPK regulates mammary milk protein synthesis: mTORC1 is the master regulator that controls milk protein synthesis in the mammary gland through 4EBP1 and S6K1. AMPK directly inhibits mTORC1 through the phosphorylation of raptor at Ser 792. AMPK also decreases mTOR activity through the phosphorylation of TSC2 (a negative mTORC1 regulator) at Ser1345. The genes encoding a_s1_-casein (CSN1S1), β-casein (CSN2) and κ-casein (CSN3) are also decreased with the activation of AMPK. 4EBP1, eukaryotic initiation factor 4E binding protein 1; AID, auto-inhibitory domain; CBS, cystathionine-beta-synthase; eEF2, eukaryotic elongation factor 2; eEF2K, eukaryotic elongation factor 2 kinase; eIF4A, eukaryotic initiation factor 4A; eIF4E, eukaryotic initiation factor 4E; eIF4G, eukaryotic initiation factor 4G; Raptor, regulatory associated protein of TOR; Rheb, ras homolog enrichedin brain; S6K1, s6 kinase 1; TSC2, tuberin 2; mTOR, mammalian target of rapamycin; mTORC1, mammalian target of rapamycin complex 1; mLST8, mammalian ortholog of LST8.

mTORC1 is a master regulator of anabolic and catabolic metabolism in cells (Howell and Manning, 2011). More specifically, mTORC1 participates in the regulation of gene transcription (Mayer and Grummt, 2006), protein translation (Mamane et al., 2006), and apoptosis (Thedieck et al., 2013). Eukaryotic initiation factor 4E binding protein (4EBP1) and ribosomal protein S6 kinase 1 (S6K1), which mainly regulate protein translation, are two independent downstream regulators of mTORC1 (Kim and Guan, 2019). Two independent pathways have been previously reported to inhibit mTOR activity through AMPK activation (Figure 4). First, AMPK inhibits TSC2 (Tuberin 2, which is a negative regulator of mTORC1) through the phosphorylation of Ser1345 (Inoki et al., 2006). Second, AMPK inhibits Raptor (a component of mTORC1) through the phosphorylation of Ser792 (Carling et al., 1987). In the mammary gland, AMPK is also reported to regulate the phosphorylation of TSC2 (Inoki et al., 2006) and Raptor (Burgos et al., 2013).

The other potential downstream target of AMPK in the regulation of protein synthesis is eukaryotic translation elongation factor 2 kinase (eEF2K), which is a negative regulator of protein elongation (Leprivier et al., 2013). As a downstream target of eEF2K, eEF2 is significantly increased during the lactation period in the bovine mammary gland (Bionaz and Loor, 2011). This observation suggests that eEF2K is a crucial regulator of milk synthesis during lactation.

### Milk Lactose

In the mammary gland, 55–70% of glucose is used for lactose synthesis (Guinard-Flament et al., 2006). Glucose acts as a substrate and provides energy for lactose synthesis. Compared with studies regarding AMPK signaling during milk protein and fat synthesis, studies regarding AMPK signaling during lactose production are limited. This gap in the literature might be because the mammary gland is the only model in which lactose synthesis can be studied. In addition, technical challenges make it difficult to induce high levels of lactose synthesis in the mammary epithelial model *in vitro*. The mammary lactose synthesis pathway has been previously summarized (Zhang S. et al., 2018). Briefly, glucose and UDP-galactose (derived from glucose) are synthesized into lactose in the Golgi bodies. Some evidence indicates that lactose synthesis is inhibited by AMPK activation. AMPK activation (stimulated by A-769662) is found to decrease the rate of lactose synthesis in the bovine mammary gland (Huang et al., 2020). Interestingly, activated AMPK increases GLUT1 mRNA expression and glucose uptake in the goat mammary gland (Zhang et al., 2011). AMPK-induced increased GLUT1 expression occurs through the phosphorylation of thioredoxin-interacting protein (TXNIP) at Ser308 (Wu et al., 2013). Although glucose is a critical source for lactose synthesis, the increase in GLUT1 does not indicate increased mammary lactose synthesis. Under energy deprivation conditions, increased cellular glucose is directly used to generate energy to maintain basic cellular function rather than to synthesize lactose. The detailed mechanism by which AMPK regulates the enzymes involved in lactose synthesis is still unclear and requires further research.

## AMPK Regulates Mammary Gland Development

The Janus kinase (Jak)-signal transducer and activator of transcription (Stat) signaling pathway is critical for mammary gland development (Hennighausen and Robinson, 2001; Wagner et al., 2004). Either glucose or amino acid deprivation activates AMPK and simultaneously decreases the Jak2/STAT5 signaling pathway in bovine mammary epithelial cells (Zhang M. et al., 2018). Initially, it was proposed that Jak2/STAT5 is involved in alveologenesis and mammary epithelial cell maintenance (Wagner et al., 2004; Caffarel et al., 2012). Recently, Jak2/STAT5 was also found to regulate milk protein synthesis through the regulation of casein translation (Yang et al., 2015). However, the evidence supporting a direct connection between AMPK and Jak2/STAT5 is still unclear and requires more study in the future.

## AMPK Functions Beyond Milk Synthesis and Mammary Gland Development

**FIGURE 5:**
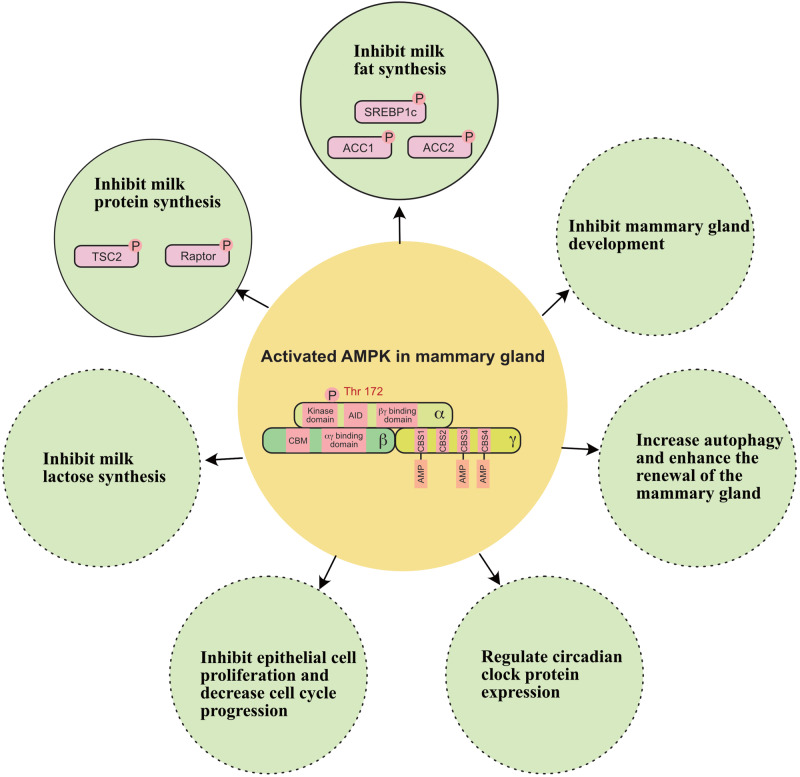
A working model of how AMPK regulates milk secretion and mammary gland function. ACC1, acetyl-coA carboxylase 1; ACC2, acetyl-coA carboxylase 2; SREBP1c, sterol regulatory element binding protein-1c; TSC2, tuberin 2. Solid circle indicates this function has been widely verified in the mammary gland. Dashed circle indicates this function still needs to be verified in the mammary gland.

In addition to regulating milk production, recent evidence indicates that AMPK is involved in the regulation of other biological functions of mammary glands (Figure 5); these other biological functions include: (1) inhibiting epithelial cell proliferation and decreasing cell cycle progression in the mammary gland (Wang et al., 2019); (2) regulating circadian clock protein expression in the mammary gland (Hu et al., 2020); (3) relieving oxidative stress in the mammary gland (Guo et al., 2020); and (4) increasing autophagy and enhancing the renewal of the mammary gland (Li et al., 2020).

## Conclusion and Outlook

To date, AMPK appears as a critical sensor in the mammary gland that detects nutrient fluctuations and environmental changes. Energy deprivation and heat stress are two major elements involved in the activation of AMPK in the mammary gland. AMPK regulates milk production (fat, protein, and lactose) and mammary gland biology (development and proliferation) through the regulation of transcription and post-translational modifications.

To the best of our knowledge, the existing evidence regarding the effects of the AMPK pathway on the mammary gland was mainly obtained under conditions of energy deprivation (amino acid or glucose deprivation). It is important to understand the function of AMPK and its related signaling pathways in the mammary gland using tissue-specific knockout mice. Future studies using AMPK-specific agonists and antagonists may also extend our understanding of AMPK signaling in the mammary gland. To date, most research studying normal mammary gland were performed in cows, mice and sows, rather than humans. Thus, it is of great importance to investigate the functions of AMPK in humans.

The most direct way to inactivate AMPK is to increase energy intake. However, according to production experience, it is very challenging to increase energy intake during the lactation period in mammals due to the limited gut volume. Thus, it would be interesting to determine whether an AMPK antagonist could rescue the decrease in milk production caused by energy deprivation and/or heat stress. The identification of novel nutritional and pharmacological inhibitors of AMPK, which can be widely and safely used as food or feed additives in mammalian diets, remains a major challenge.

## Author Contributions

SZ initiated the idea, the scope, and the outline of this review. ZW, MT, JC, JH, and FC studied and analyzed all of the publications cited in this manuscript and were involved in the manuscript preparation. WG conducted the final editing and proofreading. All authors read and approved the final manuscript.

## Conflict of Interest

The authors declare that the research was conducted in the absence of any commercial or financial relationships that could be construed as a potential conflict of interest.

## Abbreviations

4EBP1: eukaryotic initiation factor 4E binding protein 1
ACC: acetyl-coA carboxylase
ADP: adenosine diphosphate
AMP: adenosine monophosphate
AMPK: AMP-activated protein kinase
ATGL: adipose triglyceride lipase
ATM: ataxia telangiectasia mutated
ATP: adenosine 5 triphosphate
CaMKK β: Ca^2+/^calmodulin-activated protein kinase kinase- β
CPT1: carnitine palmitoyltransferase 1
CSN1S1: Casein Alpha S1
DMI: dry matter intake
eEF2K: eukaryotic translation elongation factor 2 kinase
FA: fatty acid
FABP3: fatty acid-binding protein 3
FAS: fatty acid synthase
GLUT4: glucose transporter 4
Jak: Janus kinase
LKB1: liver kinase B1
LXR α: liver X recepter α
mTORC1: mammalian target of rapamycin complex 1
PIKK: phosphoinositide 3 kinase-like kinase
PPAR γ: peroxisome proliferators-activated receptors γ
ROS: reactive oxygen species
S6K1: ribosomal protein S6 kinase 1
SCD: stearoyl-CoA desaturase
SREBP1c: sterol regulatory element-binding protein 1c
Stat: signal transducer and activator of transcription
TAG: triacylglycerol
TBC1D1: TBC1 domain family member 1
TSC2: Tuberin 2
TXNIP: thioredoxin-interactingprotein.
